# Relevance of electrical current distribution to the forced flow and grain refinement in solidified Al-Si hypoeutectic alloy

**DOI:** 10.1038/s41598-018-21709-y

**Published:** 2018-02-19

**Authors:** Y. H. Zhang, Y. Y. Xu, C. Y. Ye, C. Sheng, J. Sun, G. Wang, X. C. Miao, C. J. Song, Q. J. Zhai

**Affiliations:** 1 State Key Laboratory of Advanced Special Steels, Shanghai Key Laboratory of Advanced Ferrometallurgy, School of Materials Science and Engineering, Materials Genome Institute, Shanghai University, Shanghai, 200072 P.R. China; 2 Centre of Advanced Materials Processing and Manufacturing (AMPAM), The University of Queensland, St Lucia, QLD 4072 Australia; 3 School of Materials Science and Engineering, University of Science and Technology Liaoning, Liaoning, 114051 P.R. China

## Abstract

Significant grain refinement in cast metals can be achieved through the application of electric currents during the solidification process. The present paper investigates the distribution of electric currents on the grain size of solidified Al-7wt.%Si alloy under the application of electric current with constant parameters flowing through two parallel electrodes into the melt within a cylindrical mould. The distribution of electric current was controlled by applying an electrical insulation material coating, boron nitride (NB), to the sidewall of the electrodes. Experimental results showed that the employment of these insulated electrodes can reduce grain size in comparison with the reference case of electrodes without BN coating. Flow measurements were performed in Ga-20wt.%In-12wt.%Sn liquid metal. Higher intensity forced flow occurred when the sidewall of the electrodes was insulated. In order to understand the underlying mechanism behind the stronger forced flow, corresponding numerical simulations were performed to reveal the distributions of the electric current, magnetic field, Lorentz force, and the resultant forced flow. The results achieved indicate that the mechanism of grain refinement driven by electric current is dendrite fragmentation induced by forced flow. In addition, a novel approach to enhance the grain refinement without additional input of current energy was developed.

## Introduction

Grain refinement during the metal solidification stage plays a vital role in achieving a high level of the uniformity in the microstructure and properties of castings, and in improving the downstream rolling/extrusion processability of castings. A large number of investigations have revealed that the grain structure can be refined effectively through the application of external energy fields in forms such as electric currents^1,2^, pulse magneto-oscillation^3^, electromagnetic stirring^4^, electromagnetic vibration^5,6^ and ultrasonic vibration^7,8^, to the inoculation based technology.

It has been discovered that the passage of an electric current through a solidifying metal can lead to grain refinement owing to induced electromagnetic fields and their related effects. Solidification under the application of a current field has been attracting considerable interest both from academic researchers and industry for over 4 decades due to its high effectiveness at improving the metal microstructure, and independency to the alloy chemistry^9,10^. In particular, even lower electric consumption and more significant grain refinement can been realised through the implementation of electric current pulse (ECP), in which current is discharged in very short time periods from a capacitor bank with extremely high voltage of up to 10^3^ volts and conducted into melt through two electrodes^11^. Under ECP, the solidified structure can be modified from dendritic to globular grains accompanied by a distinct reduction in grain size, ascribed to the dendrite fracture caused by the electric-current-induced strong shear stresses. Significant grain refinement has been achieved with ECP in a broad range of metals and their alloys including Zn-^12^, Al-^13,14^, Mg-^15^, Fe-^16^ based alloys, indicating its excellent applicability to different metals and their alloying systems.

Numerous hypotheses regarding the grain refinement mechanisms with consideration of electric currents have been proposed, including heterogeneous nucleation^17,18^, crystal rain^13^ and dendrite fragmentation^2^. It has been reported that the heterogeneous nucleation under the application of electric current can be effectively stimulated, owing to the free energy minimisation provoked by the application of electric current^17,18^. More recently, the electric-current-induced free energy minimization for crystal nucleation has been considered as a driving force for the finer microstructure generated^19^, rotation of disk-like particles^20^ and motion of micro-particles by pulsed electric current^21^. Through an experimental study involving commercial pure aluminium solidifying under the configuration of a pair of vertically arranged parallel electrodes immersed into the melt through free surface, Li *et al*. have proposed a crystal rain mechanism to explain the grain refinement occurring^13^. It is believed that a thin layer of melt will solidify in the early stage of the melt solidification at the top free surface of the melt, which is exposed directly to the air, and a large number of nuclei from the top surface can be detached, resulting in the crystal rain being influenced by the electromagnetic force.

The electric-current-induced forced convection flow and its effect on the solidification of metals was recently investigated^2,22^. Through an experimental study, Räbiger *et al*.^2^ have demonstrated that a strong global forced flow was induced by applying electric currents in the melt under the configuration of two parallel electrodes immersed from the free surface into the electrically conducting melt. Furthermore, the corresponding numerical simulations showed the forced convection was attributed to the induced electromagnetic force, which was confined to a narrow region beneath two electrodes. When higher intensity flow was generated, finer grains were observed in the Al-7wt.%Si alloy. In addition, the experimental results indicated that almost identical equiaxed structures were observed under the same flow intensity caused by ECP and travelling magnetic field (TMF). It is widely accepted that the TMF-induced forced flow plays a key role in the grain refinement of solidified alloys^23,24^. It is therefore convincing to conclude that the forced flow would be the key driving force behind the grain refinement of alloys driven by ECP. It is most likely that dendrite fragmentation is one of the main mechanisms for grain refinement resulting from the application of electric current during metal solidification, due to the fact that forced flow induced temperature and solute fluctuations trigger remelting of dendrite arms to produce dendrite fragmentation.

In order to clarify the effect of these three potential mechanisms, current study investigates the influence of the electric current distribution on the forced flow as well as the resulting grain refinement under the application of the same electric current parameter. The configuration of two parallel electrodes immersed from the free surface into the melt was employed to conduct electric current in two ways. The reference is the electric current flowing through the lateral surface of electrodes into the free surface of melt, whereas the other is only the electric current flowing through the bottom of electrodes into the bulk melt by coating of an electrical insulation material, boron nitride (BN), on the lateral surface of the electrodes.

## Results

### Grain structure of cast sample

**Figure 1:**
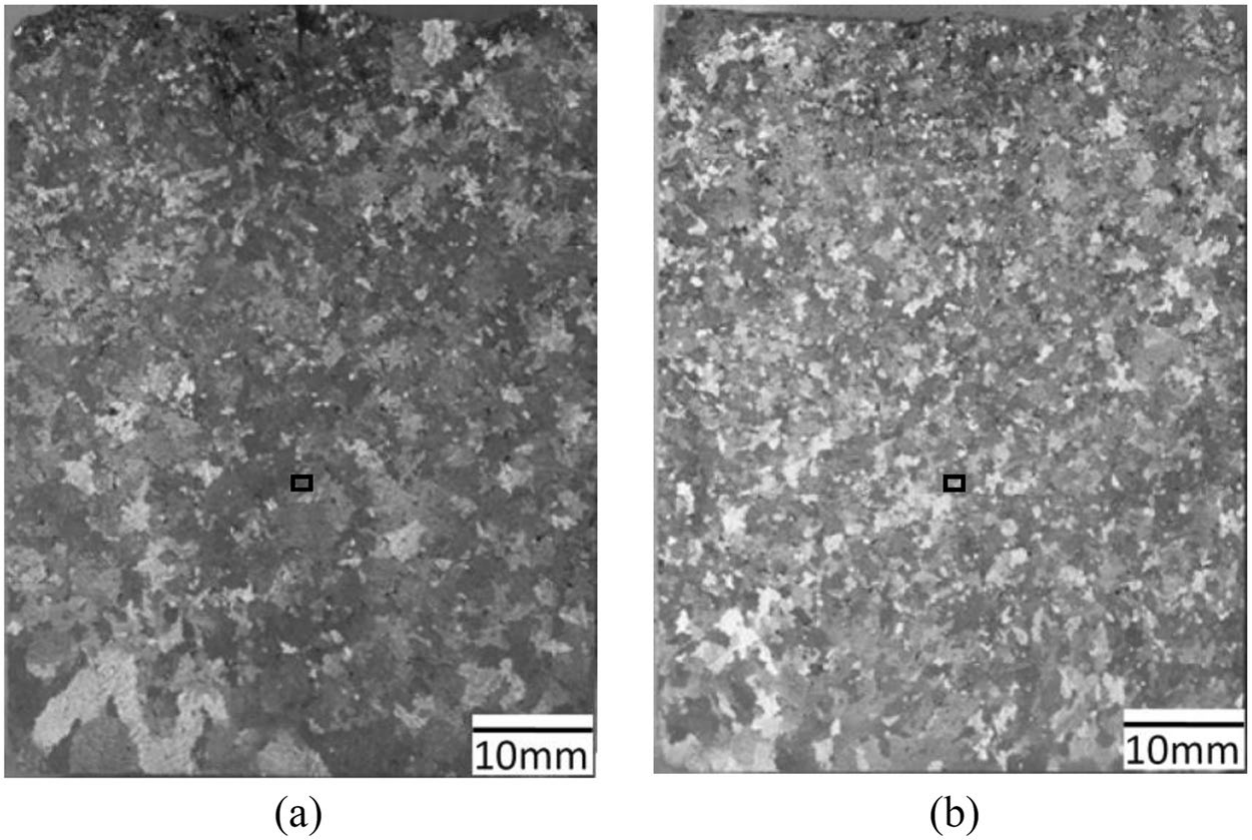
Macrostructures of Al-7wt.%Si alloy under the impact of direct current: (**a**) electrodes without BN, (**b**) electrodes sidewall with BN.

**Figure 2:**
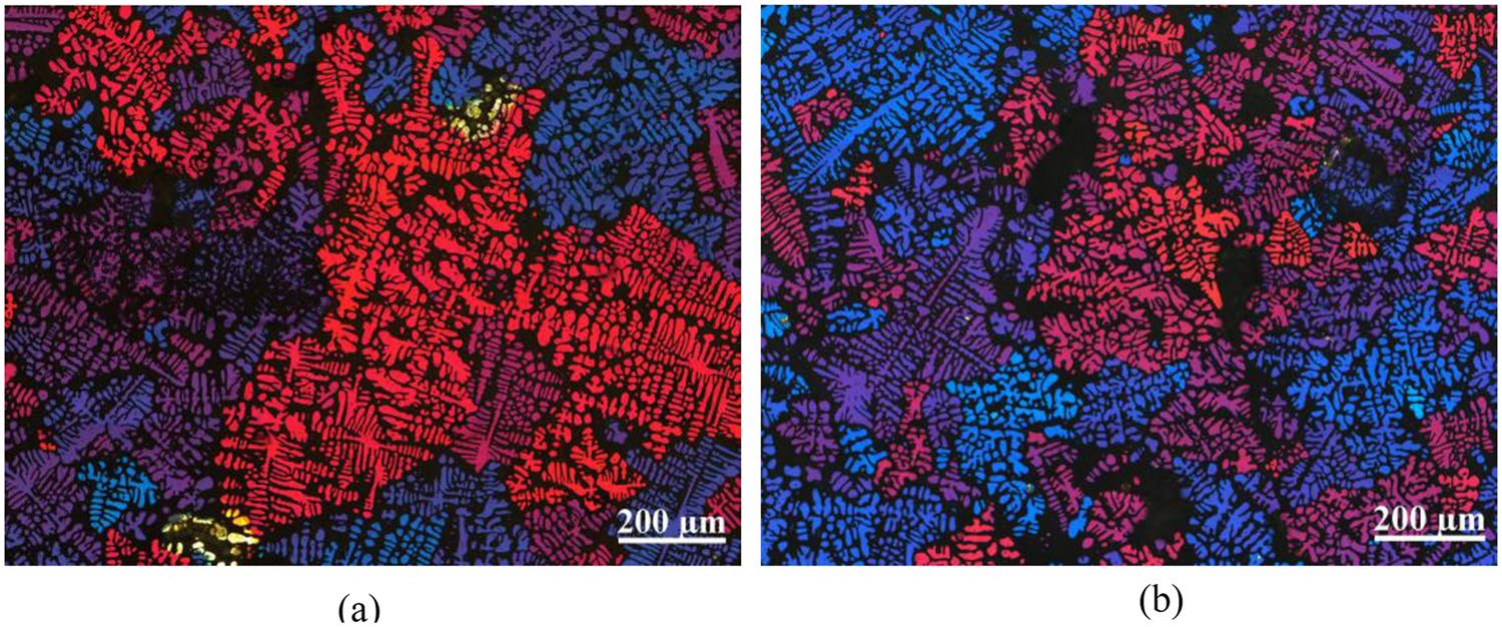
Microstructures of Al-7wt.%Si alloy under the impact of direct current (observed under the polarized light mode): (**a**) electrodes without BN, (**b**) electrodes sidewall with BN.

Figure 1 shows the longitudinal macrostructures of solidified Al-7wt.%Si alloy under the application of a direct current of *I*_*DC*_ = 152 A conducting through the two kinds of electrodes. In both of the two solidified samples, it was found that a large quantity of refined equiaxed grains were produced in comparison with the solidified macrostructure of sample without electric current achieved in our previous study^2^. As shown in Fig. 1, the equiaxed grains were almost homogeneously distributed throughout the whole samples. However, it can be observed that larger grains with a grain size of 887 ± 143 μm were produced in the reference sample treated by DC using electrodes without the BN coating as shown in Fig. 1a. In contrast, as shown in Fig. 1b, finer grains with a grain size of 606 ± 64 μm were generated in the case of when that the same electric current was only allowed to conduct through the bottom surface of the BN coated electrodes into the solidifying sample. The variation in grain size found in both samples indicates that the different electrode conditions, as well as the electric currents distributions, have a significant effect on the grain refinement of the solidified sample, even though the same geometry and electric current parameters were applied. Figure 2 presents the corresponding microstructures observed using the polarized light mode. As before, finer grains with dendrite morphology were observable in the Al-Si alloy treated using electric current flowing through the bottom of electrodes.

### Forced flow

**Figure 3:**
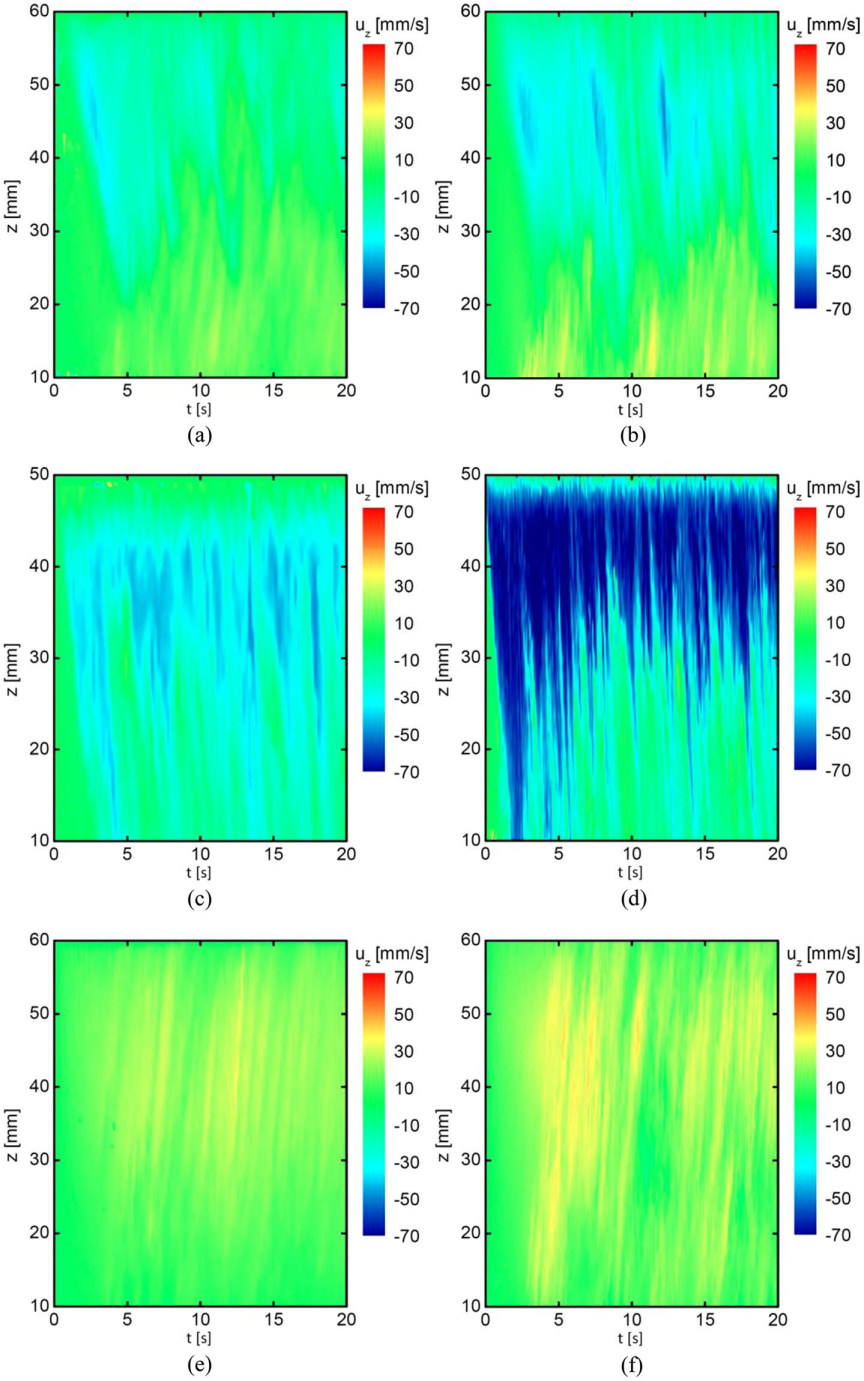
Influence of electric current distribution on the vertical velocity measured at three positions (see Fig. 7b) under the applied direct current of *I*_*DC*_ = 152 A: electric current flowing through sidewall of electrodes: (**a**) P1, (**c**) P2, (**e**) P3; through bottom surface of electrodes: (**b**) P1, (d) P2, (**f**) P3.

Flow measurements were performed in a Ga-20wt.%In-12wt.%Sn liquid metal to investigate the influence of electric current distribution on the forced flow. Figure 3 presents the vertical velocity measured at three locations under the conditions of an applied current of *I*_*DC*_ = 152 A flowing through the sidewall and bottom surface of the electrodes respectively. The same flow pattern can be observed under both of these two different electric current distributions. The flow direction at the cylinder axis was downward in the upper part and upward in the bottom part, as shown in Fig. 3a,b. Underneath the electrodes, a downstream flow was generated as revealed in Fig. 3c,d. The velocity measurement from the sidewall of the plane perpendicular with the plane containing the two parallel electrodes showed an upward flow as shown in Fig. 3e,f.

Although the same flow pattern was generated, the flow intensity inside the melt is obviously stronger at all positions when the sidewall of the electrodes was coated by BN to confine the electric current to flow only through the bottom surface of the electrodes. Particularly, at the measurement location beneath the electrodes, the largest vertical velocity was only about 40 mm/s in the reference case of the electrodes without BN in Fig. 3c, whereas the corresponding value was at least as high as 70 mm/s for the case of the sidewall of electrodes with BN in Fig. 3d. The presented results indicate that the higher flow intensity, as well as finer grain structure, can be achieved by controlling the electric current distribution, even if the employed electric current parameter is the same.

**Figure 4:**
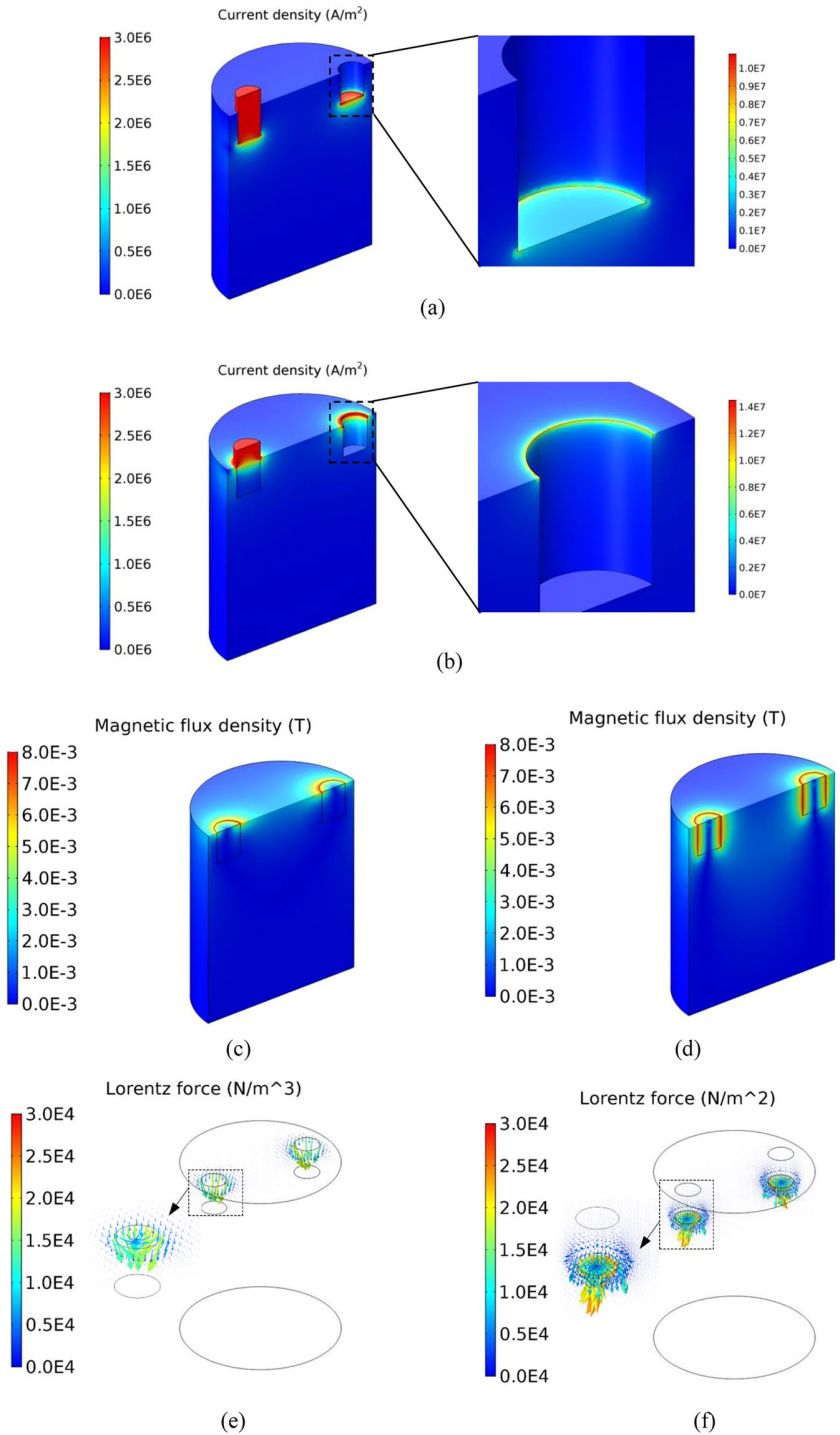
Numerically simulated electric current, magnetic field and Lorentz force distributions of direct current (152 A) in Ga-20wt.%In-12wt.%Sn alloy melt for the situation of electrodes without insulated material (**a**) electric current, (**c**) magnetic field, (**e**) Lorentz force; with insulated material (**b**) electric current, (**d**) magnetic field, (**f**) Lorentz force.

In order to understand the electric current induced melt flow in a cavity of a cylindrical mould, corresponding numerical simulations were performed to reveal the distribution of the electric current, the self-induced magnetic field and the resulting Lorenz force inside the bulk melt. The numerical results showed that the parallel electric currents flowing through the electrodes immediately spread into the melt as shown in Fig. 4a,b. The maximum current density of about 1 × 10^7^A/m^2^ was achieved and dramatically reduced by a factor of 10 to 1 × 10^6^ A/m^2^ during the spreading process. A significant concentration of electric current can be obtained at the location where the electric current runs into the melt from the electrodes in both cases. In the reference case of the electrodes sidewall without BN, the electric current is concentrated in the region of the electrodes sidewall, adjacent the free surface. In comparison, the highest electric current intensity is generated within the narrow region beneath the electrode’s bottom surface for the case of electrodes with the electrically insulated sidewall.

**Figure 5:**
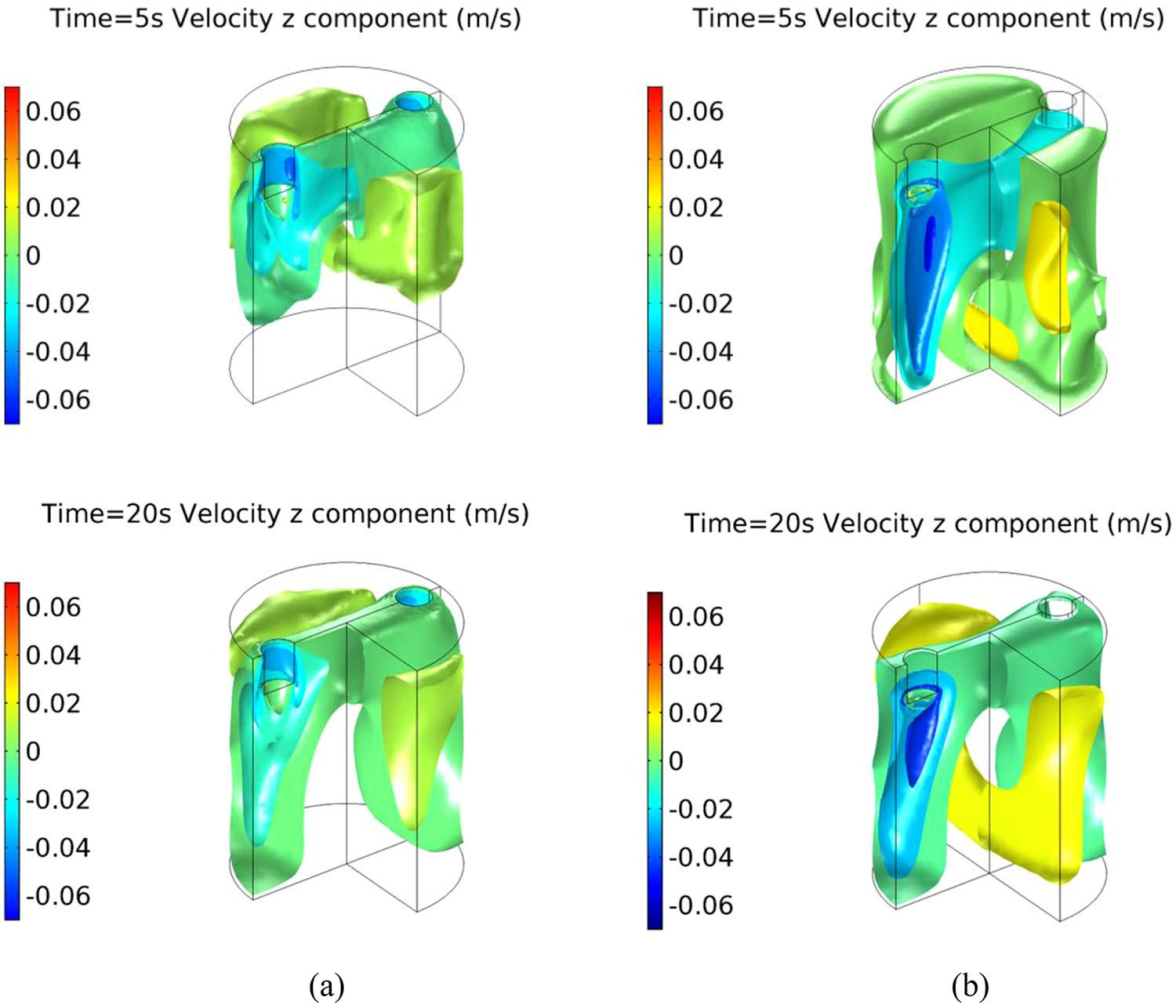
Numerically simulated forced flow induced by direct current (152 A) in Ga-20wt.%In-12wt.%Sn alloy melt for the situation of electrodes (**a**) without and (**b**) with insulated material.

As shown in Fig. 4c–f, the induced magnetic field and the resulted Lorenz force have a similar trend in terms of their distribution patterns. Both the intensities of magnetic field and Lorenz force reach their maximum at the narrow region and then severely decrease in the bulk melt owing to the widening of the electric current. In particular, the strong Lorenz force distributed at the small domain has an almost downward direction due to the interaction between the spreading electric current and its self-induced magnetic field. As a result, two strong downward flow jets were generated, causing a global forced flow inside the bulk melt. The simulated flow pattern and flow intensity are in good agreement with the measured forced flow as shown in Figs 3 and 5.

Since both the magnetic field and Lorenz force are induced by the electric current, it should be noticed that the concentrated region of magnetic field and Lorenz force can be influenced by coating the electrical insulation materials on the lateral surface of electrodes, as shown in Fig. 4. The strongest Lorenz force is located at the lateral surface of the electrodes just underneath the free surface of bulk melt in the reference case, whereas the concentrated region of Lorenz force is below the bottom surface of the electrodes for the case of electrodes where the sidewall is electrically insulated by BN. According to the simulated results, the stronger and more concentrated Lorenz force is achieved in the sample when using insulated electrodes rather than the electrodes without BN as shown in Fig. 4c. As a consequence, stronger forced flow is generated for the case of the insulated electrodes, as shown in the experimental and simulated results (see Figs 3 and 4).

## Discussion

The metallographic images of the as-cast grain structure have shown that a smaller grain size can be achieved by confining the electric current such that it only flows through the bottom of the electrodes into the melt, even though the same parameters are employed for the electric currents. The numerical results further prove that a forced melt flow with higher intensity is generated under the situation when the electric current flows through the bottom surface of the electrodes rather than the sidewall, which is confirmed by the corresponding flow measurements. The results convincingly demonstrate that the grain refinement of solidified alloys is driven by the intensity of the electric-current-induced forced flow. The most likely grain refinement mechanism is the dendrite fragmentation. The induced forced flow causes localized fluctuations of both temperature and solute concentration in order to provoke the occurrence of dendrite fragmentation. The stronger forced flow significantly promotes higher dendrite fragmentation rates as well as finer grains. This is the reason why the finer grains are produced in the current experimental condition, since the stronger forced flow is generated by controlling the distribution of electric current. Moreover, it has been recognized that the solidified shell with dendrite structure is firstly formed on the wall of mould during the initial solidification period. The concentrated Joule heating and Lorentz force as shown in Fig. 4 can not directly applied on the solidified shell in our employed experiment configurations. It means that the contribution of Joule heating and Lorentz force to provoke the dendrite fragmentation can be ignored, which has been confirmed by the related research in ref.^2^.

However, the other two mechanisms, heterogeneous nucleation promoted by electric-current-induced free energy minimization and crystal rain mechanisms, should be clarified. According to the proposed free energy minimization mechanism, the change in the free energy under the application of electric current ΔG_EC_* is given by^17^:

1 $${\rm{\Delta }}{G}_{EC}^{\ast }={\rm{K}}{{\bf{J}}}^{2}\zeta {V}_{n}$$

where *K* is the constant of material, **J** is the electric current density, *V*_*n*_ is the volume of nucleus, *ζ* is calculated by^17^:

2 $${\rm{\zeta }}=\frac{{\sigma }_{0}-{\sigma }_{n}}{{\sigma }_{n}+2{\sigma }_{0}}$$

where *σ*_0_ is the electric conductivity of the melt, and *σ*_*n*_ is the electric conductivity of the nucleus. Since the electric conductivity of the melt is less than that of the nuclei (solid phase) in Al-7wt.%Si alloy, the *ΔG*_EC_^*^ has a negative value, which means the free energy ΔG_EC_* decreases under the application of electric current, reducing the nucleation energy barrier. According to equation 1, the reduction quantity of free energy, as well as the resulted grain size, only depends on the electric current density **J** inside the melt. In this study, the maximum electric current density in the concertation region is 1.4 × 10^7^A/m^2^ in the case of electrodes without BN, whereas a little lower maximum electric current density with value of 1 × 10^7^A/m^2^ was generated when the sidewall of electrodes was electrically insulated as shown in Fig. 4a,b. It indicates that the smaller grain size would be generated in the solidified sample in the case of electrodes without BN. However, the larger grain sizes achieved in our experimental results means that the free energy minimization mechanism would be not the driving one in our case. In addition, due to the fact that the case of electrodes without BN was frequently employed in the previous studies^13,21^, the crystal rain mechanism is proposed in which a solidified shell is formed at the free surface, and crystal rain from the shell can then be promoted by the concentrated electric current at the free surface (see Fig. 4a). However, the larger grain size is generated for the case of electrodes without BN in comparison with the case of electric currents concentrated at the region inside the bulk melt due to the lateral surface of electrodes with the BN coating. Therefore, this indicates that the crystal rain mechanism is not the one driving grain refinement as a result of the electric current.

Moreover, the knowledge presented in the present paper has demonstrated that it is a feasible approach to optimize the grain refinement by controlling the distribution of the electric current. Investigating the effect of the electric current distribution on the grain refinement of alloys is highly attractive for industry on account of the fact that the finer grains can be achieved without additional energy input. Here we have demonstrated a candidate method to control the electric current distribution by means of coating an electrical insulation material on the electrodes. This insight presents a clue to develop other alternative methods for the improvement of the electric current distribution, as well as the grain refinement, without more energy consumption.

## Methods

### Solidification experiment

**Figure 6:**
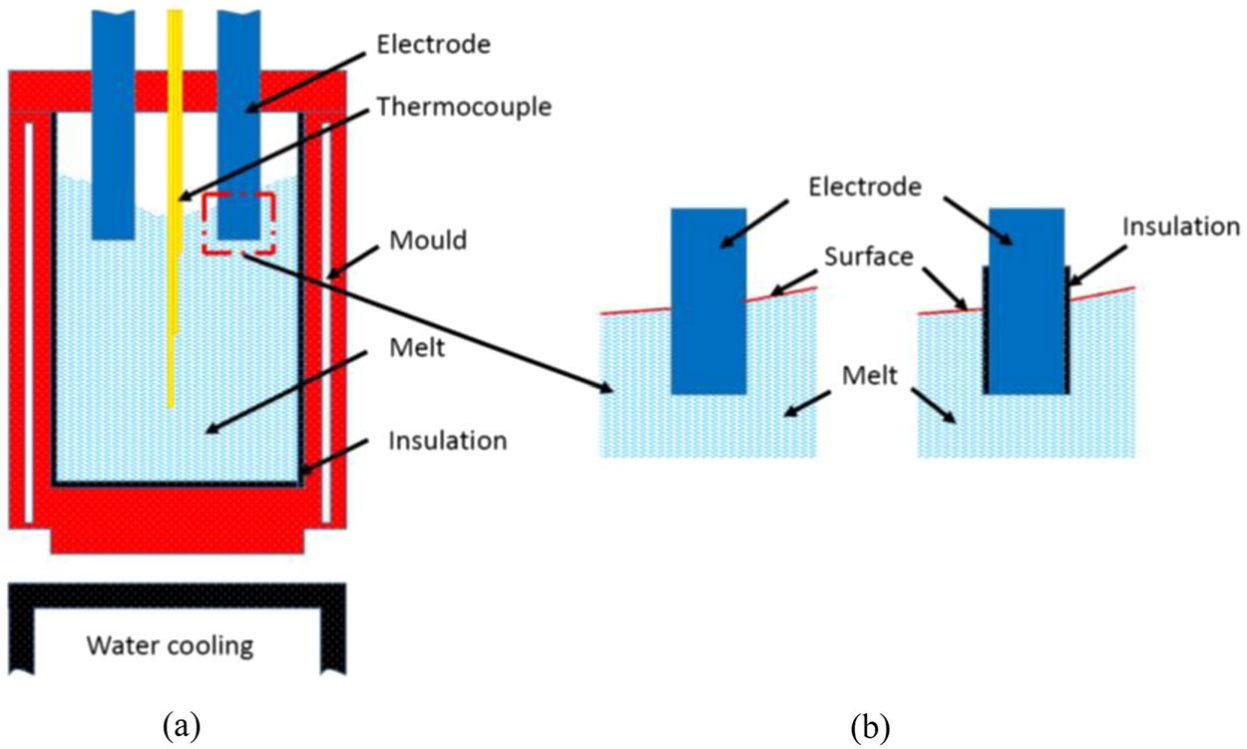
Schematic views of (**a**) solidification experimental setup and (**b**) electrodes (sidewall with and without electrical insulation material, boron nitride (BN)).

Figure 6a schematically shows the experimental setup. A double-walled stainless steel mould with an inner diameter of 50 mm was located on the surface of a copper cooling platform to allow for vertically directional solidification. The temperature of the cooling platform was maintained at 20 °C through running water beneath of the platform. In order to avoid electric current flowing into the mould, the inner wall of the mould was coated by an electrical insulation material, Boron Nitride (BN), which has been widely employed as releasing agent to coat on the wall of mould. Two parallel stainless steel electrodes with a diameter of 8 mm and a distance of 36 mm between each other were immersed into the melt with a depth of 10 mm. The power supply, pe86CWD (plating electronic), was employed to generate direct current. K type thermocouples with an INCONEL (trademark of INCO Alloys International, Huntington, WV) sheath, outer diameter of 1.5 mm and covered by Al_2_O_3_ tubes, was vertically located at the axis of the sample to measure temperature. Temperature were recorded through a data acquisition system, and the sampling frequency, resolution and error of the temperature measurement system was 5 Hz, 0.01 °C and ± 1 °C, respectively.

An Al-7wt.%Si hypoeutectic alloy (nominal composition) was prepared using commercially pure Al (99.99 wt.%) and pure Si (99.999 wt.%) in a clay graphite crucible using a resistance furnace. After melting, the alloy was pouring into a stainless mould with a diameter of 50 mm. The cast alloys were then cut to the same weight of 310 g for casting experiments. Alloyed samples were remelted in the double walled mould, heated to 750 °C holding for 45 mins, and subsequently cooled to 720 °C in the resistance furnace. After holding for 30 mins at 720 °C, the molten alloy with the mould was removed from the furnace and then assembled with thermocouples and electrodes, eventually transferred to the cooling platform to solidify the sample.

Electrodes without the insulation material were employed as the reference, a consideration which was adopted in previous research^13^. Such an electrode means that the electric current can flow through the sidewall of the electrode. The electrical insulation material, BN, was applied to control this electric current distribution. As the schematic view demonstrates, shown in Fig. 6b, the lateral wall of electrodes was covered with BN to limit the electric current such that it only flowed through the bottom surface of the electrodes. Both kinds of electrodes were used to perform solidification experiments using conducting DC of 152 A in the Al-7wt%Si alloy. The forced cooling of the sample through the mould bottom and the treatment by the DC were triggered at almost the same time. The application of the electric currents was powered off until the samples were cooled to the temperature of the eutectic point.

### Metallography

The solidified samples were longitudinally sectioned for metallographic analysis. One section was ground using SiC paper, etched in a solution composed of 60 mL HCl, 30 mL HNO_3_, 5 mL HF, and 5 mL H_2_O, and then directly photographed via a digital camera (Konica Minolta) for the macrostructure analysis. Selected regions in the other section were ground and polished from 6 μm to 1 μm, electro-etched in Barker’s etching reagent at 25 V, 15HZ for 120 s, and then examined under an optical microscope Axio Imager A2m (Carl Zeiss, Germany) using the polarized light for the quantitative analysis of grain size by the linear intercept method.

### Melt velocity measurement

**Figure 7:**
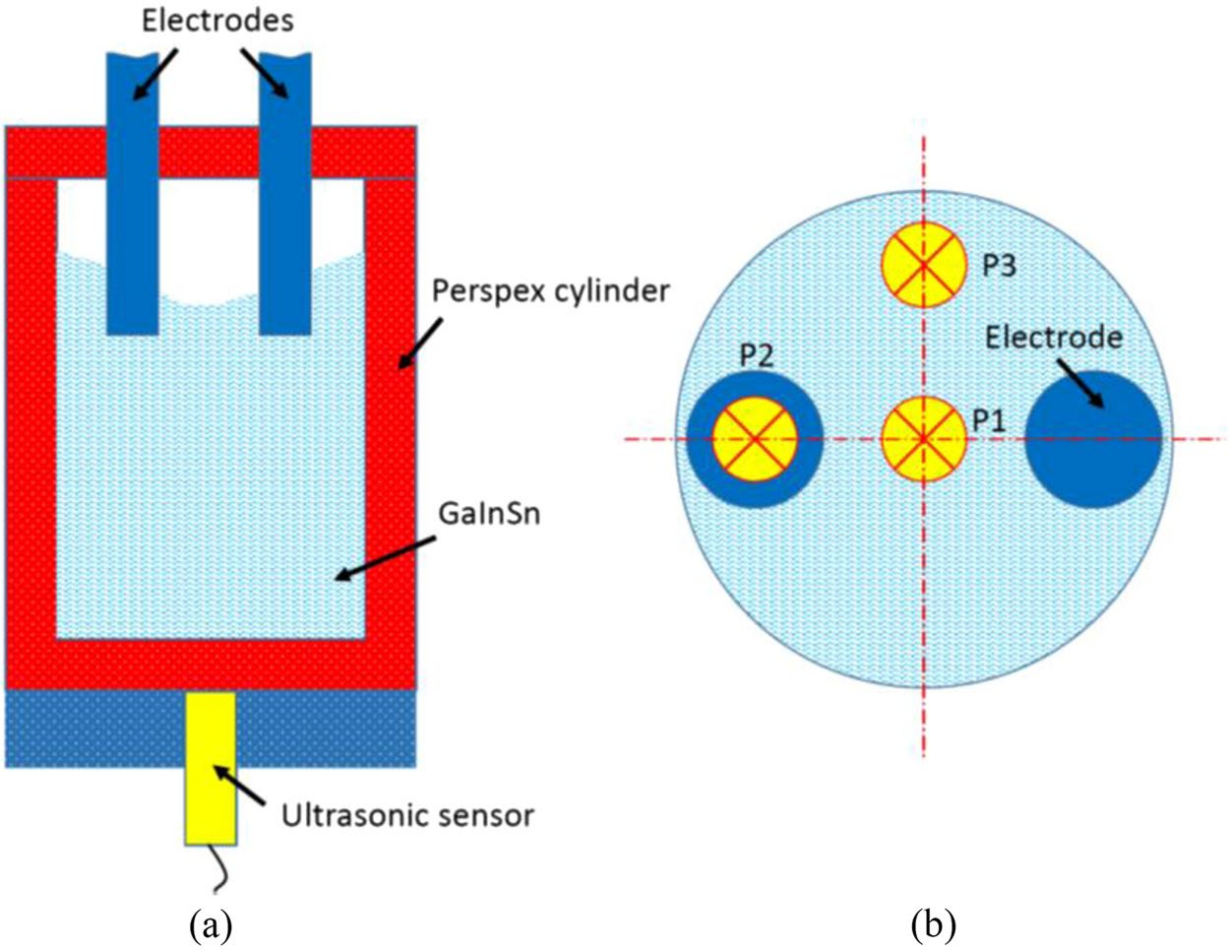
Schematic views of (**a**) flow measurement setup and (**b**) single transducer arrangement at three positions of bottom.

The ultrasound Doppler method (UDV) technique was performed to monitor the melt velocity under the application of the electric current. Since the temperature of the Al-Si melt was higher than temperature limitation of the sensor, a ternary eutectic alloy (Ga-20wt.%In-12wt.%Sn), which undergoes melting at room temperature, was used in a vessel made from perspex with the same inner size as the solidification experiment. The similar dimensionless parameter S is achieved in both Al-7wt%Si and Ga-20wt.%In-12wt.%Sn alloy melt^2^. Hence, the similar flow structure and intensity can be assumed in these two alloy melts for the same employed configuration and electric current parameter. An 8 MHz transducer (TR0805LS, acoustic active diameter 5 mm) from the DOP2000 velocimeter (model 2125, Signal Processing SA, Lausanne) was located at the three positions of the bottom along the vertical direction (see Fig. 7). The spatial resolution along the vertical direction was about 1.4 mm, and the accuracy of the velocity data can be assessed to be better than 0.15 mm/s.

### Numerical model

**Figure 8:**
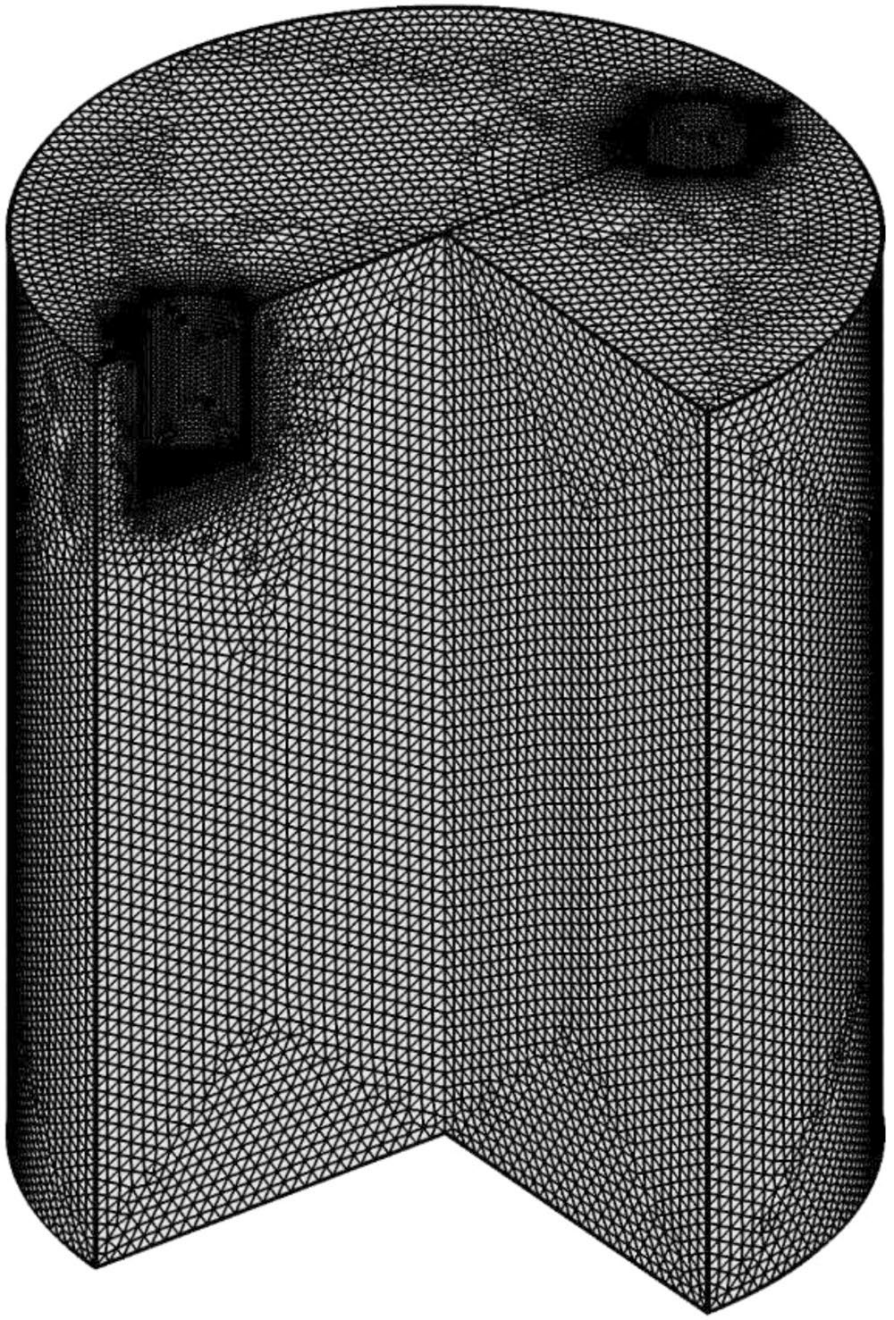
Geometry of numerical domain with meshes.

Commercial software CFX (ANSYS) was employed to numerically simulate the distributions of electric current, induced magnetic field, Lorentz force and forced flow inside the GaInSn liquid. The material properties used for this simulation are shown in Table 1. As shown in Fig. 8a, a three-dimensional domain was constructed according to the geometry and size of the flow measurement setup, and a finer grid was utilised in the region around the electrodes. The total number of elements in the mesh was 443 million.

**Table 1:** Properties of Ga-20wt.%In-12wt.%Sn liquid metal^2^.

Density (kg · m^−3^)	6360
Kinematic viscosity (ms^−2^)	0.34 · 10^−6^
Electrical conductivity (S · m^−1^)	3.27 · 10^6^

The following three boundary condition types have been assigned:

Electric field:

1. Current Inlet (top face on one of electrodes): fixed value for electric current density.
2. Current Outlet (top face on another electrode): electric potential equal to zero.
3. Current insulation (lateral face on both of electrodes and top surface of melt): zero gradient of electric current density normal to the wall.

Magnetic field:

Magnetic insulation (external layer of the air): magnetic flux density equal to zero.

Fluid field:

1. Slip wall (free surface of the fluid next to the air): slip condition was set where velocity normal to the wall equal to zero.
2. No slip wall (all rigid walls): velocity (normal or tangential component) equal to zero.

According to Ohm’s law, the electric current density **J** gives by:

3 $${\bf{J}}=\sigma ({\bf{E}}+{\bf{U}}\times {\bf{B}})$$

where σ, E, U are the electrical conductivity, the electric field and the velocity, respectively.

The magnetic field strength **B** is calculated by Biot-Savart’s law:

4 $${\bf{B}}=\frac{{\mu }_{0}}{4\pi }{\iiint }_{V}\frac{({\bf{J}}dV)\times {\bf{r}}}{{r}^{3}}$$

where μ_0_, V and **r** are the permeability of a vacuum, the volume and the vector of displacement, respectively.

Lorenz force **F** is determined from the interaction between electric current and induced magnetic field, which is defined as:

5 $${\bf{F}}={\bf{J}}\times {\bf{B}}$$

According to the magneto-hydrodynamic theory, the governing equations of fluid flow are based on the continuity equation

6 $$\nabla \cdot {\bf{U}}=0$$

and momentum equation

7 $$\rho (\frac{\partial {\bf{U}}}{\partial t}+({\bf{U}}\cdot \nabla ){\bf{U}})=-\nabla p+\mu {\nabla }^{2}{\bf{U}}+{\bf{F}}$$

where ρ, μ and p are the density, the molecular dynamic viscosity and the pressure, respectively.

## Conclusion

The current paper investigates the influence of electric current distribution on the forced flow and the resulting solidified structures of Al-7wt.%Si alloy. The experimental results showed that although the same electric current parameter of direct current is applied, finer grain size is achieved when the electric current is limited by boron nitride (BN) such that it flows only through the bottom of electrodes in comparison with the reference case without the limitation of BN. This phenomenon is due to the stronger forced flow caused in the case of electrodes with their sidewall coated with BN, as the flow measurement results clearly demonstrated. The numerical results show that the electric current is significantly concentrated at the narrow region beneath the bottom of the electrodes for the case with the electrodes sidewall coated by BN, whereas in the case without the BN coating, the electric current rapidly spreads from the lateral surface of the electrodes, adjacent the free surface, into the melt. Since the interaction of electric current and self-induced magnetic field results in Lorentz force, the corresponding simulated distribution of Lorentz force shows that concentration of the electric current underneath the bottom surface generates higher downward Lorentz force, causing a stronger downward jet to force the global flow in the bulk melt more as well as achieve a finer gain size.

Three proposed mechanisms of electric-current induced grain refinement have been clarified by the knowledge gained in this study. The most likely mechanism is the dendrite fragmentation initiated by the electric-current induced forced flow. The present research not only provides more insights into understanding the grain refinement mechanism under the application of electric current, but also presents a potential approach to achieving a reduction in grain size without an additional input of energy.
